# Patient Engagement as a Component of a Learning Healthcare System: A case study using small area rate variation research in Nova Scotia, Canada

**DOI:** 10.23889/ijpds.v1i1.305

**Published:** 2017-04-19

**Authors:** Adrian Levy, George Kephart, Laura Dowling, Jonathan Dyer, Frederick Burge, Frank Atherton, Adrian MacKenzie, Leslie Anne, Juergen Krause, Ted McDonald

**Affiliations:** Maritime SPOR SUPPORT Unit; Dalhousie University; SPOR Primary and Integrated Health Care Innovations Network; Nova Scotia Department of Health and Wellness; University of Prince Edward Island; University of New Brunswick

## Objectives

The objective was to develop a framework for incorporating patient engagement into administrative health database research. In an examination of variation in high-cost health service use using administrative data, patient experience was incorporated as an additional source of knowledge to inform evidence-informed policy making in a learning healthcare system framework.

## Method

The study described variation in the rate of high-cost use by area within Nova Scotia, Canada, and isolated local factors contributing to the rate of high-cost use to inform targeted intervention development. Regression analysis was used to determine where the rate of high-cost use was driven by known contributors, such as demographics or disease patterns. Peer-leaders from provincial chronic disease management programs (Patient Navigators) were recruited as study team members. They were invited to help describe their collective experience of patient-based factors that may contribute to high-cost use, including access to care and multi-morbidity in their regions.

## Results

The outcome of this `proxy' patient engagement was measured by the extent to which the input from the Patient Navigators influenced study protocol, interpretation of results and communication of findings. The patient voice helped describe the extent of variation, contextualize the findings, and suggested additional contributory factors not revealed by the analysis of administrative health data. For example, the Patient Navigators described regional discrepancies in available services for managing chronic disease and variation in the approach to discharge planning. In this way, the patient experience was incorporated to attempt to explain rates of high-cost use in areas that could not be explained by known contributors. Further, patient experience with travel distance to receive care and alternate levels of care helped to generate questions for future research.

## Conclusion

Patient experience is an invaluable input into health research that contributes to health system planning. This research incorporated `proxy' patient experience to produce evidence to inform targeted interventions aimed at reducing the rate of high cost-users. The identified areas where focused interventions or reforms could yield material benefits for efficient delivery of health care.

